# Outcomes With Multidisciplinary Cardiac Rehabilitation in Post-acute Systolic Heart Failure Patients—A Retrospective Propensity Score-Matched Study

**DOI:** 10.3389/fcvm.2022.763217

**Published:** 2022-04-12

**Authors:** Shyh-Ming Chen, Lin-Yi Wang, Mei-Yun Liaw, Ming-Kung Wu, Po-Jui Wu, Chin-Ling Wei, An-Ni Chen, Tsui-Ling Su, Jui-Kun Chang, Tsung-Hsun Yang, Ching Chen, Cheng-I Cheng, Po-Cheng Chen, Yung-Lung Chen

**Affiliations:** ^1^Section of Cardiology, Department of Internal Medicine, Heart Failure Center, Kaohsiung Chang Gung Memorial Hospital and Chang Gung University College of Medicine, Kaohsiung, Taiwan; ^2^Department of Physical Medicine and Rehabilitation, Kaohsiung Chang Gung Memorial Hospital and Chang Gung University College of Medicine, Kaohsiung, Taiwan; ^3^Department of Psychiatry, Kaohsiung Chang Gung Memorial Hospital and Chang Gung University College of Medicine, Kaohsiung, Taiwan; ^4^Department of Nursing, Heart Failure Center, Kaohsiung Chang Gung Memorial Hospital, Kaohsiung, Taiwan; ^5^Department of Physical Therapy, Kaohsiung Chang Gung Memorial Hospital, Kaohsiung, Taiwan; ^6^Department of Occupational Therapy, Kaohsiung Chang Gung Memorial Hospital, Kaohsiung, Taiwan; ^7^Clinical Psychologist, Department of Psychiatry, Kaohsiung Chang Gung Memorial Hospital and Chang Gung University College of Medicine, Kaohsiung, Taiwan

**Keywords:** cardiac rehabilitation, multidisciplinary program, heart failure, mortality, renin-angiotensin-aldosterone system

## Abstract

**Background:**

Cardiac rehabilitation (CR) is recommended for patients with acute heart failure (HF). However, the results of outcome studies and meta-analyses on CR in post-acute care are varied. We aimed to assess the medium- to long-term impact of CR and ascertain the predictors of successful CR.

**Methods:**

In this propensity score-matched retrospective cohort study, records of consecutive patients who survived acute HF (left ventricular ejection fraction <40) and participated in a multidisciplinary HF rehabilitation program post-discharge between May 2014 and July 2019 were reviewed. Patients in the CR group had at least one exercise session within 3 months of discharge; the others were in the non-CR group. After propensity score matching, the primary (all-cause mortality) and secondary (HF readmission and life quality assessment) outcomes were analyzed.

**Results:**

Among 792 patients, 142 attended at least one session of phase II CR. After propensity score matching for covariates related to HF prognosis, 518 patients were included in the study (CR group, 137 patients). The all-cause mortality rate was 24.9% and the HF rehospitalization rate was 34.6% in the median 3.04-year follow-up. Cox proportional hazard analysis revealed that the CR group had a significant reduction in all-cause mortality compared to the non-CR group (hazard ratio [HR]: 0.490, 95% confidence interval [CI]: 0.308–0.778). A lower risk of the primary outcome with CR was observed in patients on renin-angiotensin-aldosterone system (RAAS) inhibitors, but was not seen in patients who were not prescribed this class of medications (interaction *p* = 0.014).

**Conclusions:**

Cardiac rehabilitation participation was associated with reduced all-cause mortality after acute systolic heart failure hospital discharge. Our finding that the benefit of CR was decreased in patients not prescribed RAAS inhibitors warrants further evaluation.

## Introduction

While the positive effects of physical activity in chronic stable heart failure (HF) patients are established (1, 2), baseline ventilatory, hemodynamic, autonomic, or clinical factors that can predict the outcome of exercise training in patients with HF remain unknown (3, 4). Additionally, previous studies have demonstrated that fewer guideline directed medical therapy (GDMT) prescriptions were associated with higher 1-year all-cause mortality (5, 6). We hypothesized that baseline GDMT prescriptions may have an impact on the outcomes of post-discharge multidisciplinary cardiac rehabilitation (CR) in patients who survived acute HF. This study aimed to use real-world data to evaluate the impact of post-discharge CR on acute HF patients with reduced ejection fraction (EF). Subgroup analyses, including age sex, comorbidities, GDM prescriptions, and functional study variables based on CR, were also performed to identify plausible interaction effects.

## Materials and Methods

### Study Design

We undertook a retrospective, observational, cohort study of patients discharged from a HF center in Southern Taiwan. This study was approved by the Institutional Review Board of the Kaohsiung Chang Gung Memorial Hospital (approval number: 202001285B0) and was conducted in accordance with the Helsinki Declaration of 1975 (as revised in 1983). The need for informed consent was waived due to the retrospective nature of the study. This study was registered at ClinicalTrials.gov (identifier: NCT04838470).

### Patients

The records of consecutive patients with acute HF (left ventricular EF <40%), discharged between May 2014 and July 2019, were reviewed. The multidisciplinary HF disease management program (HFDMP) was the standard of care in the HF center of Kaohsiung Chang Gung Memorial Hospital. The HFDMP has been previously described in detail (7). Briefly, the HFDMP included dietitian, pharmacist, and psychological consultations; psychiatric assessment; HF nursing education; and exercise training conducted by a rehabilitation physiatrist. Patients who had at least one exercise session within 3 months of discharge were placed in the CR group. Patients who did not have an exercise session were placed in the non-CR group. Both the CR and non-CR groups attended the HFDMP.

Propensity scores for the patients were determined by logistic regression analysis based on age; sex; body mass index; systolic and diastolic blood pressures; heart rate; HF etiology (ischemic); having hypertension, diabetes mellitus, hyperlipidemia, or cerebrovascular disease; hemoglobin level; estimated glomerular filtration rate (Chronic Kidney Disease Epidemiology Collaboration equation, CKD-EPI); prescription of angiotensin-converting enzyme inhibitors/angiotensin II receptor blocker (ACEI/ARB), beta-blocker, mineralocorticoid receptor antagonist (MRA), angiotensin receptor neprilysin inhibitor (ARNI), and diuretics; left ventricular EF (LVEF); and atrial fibrillation. Unequal propensity score matching (PSM) for one case with a maximum of three controls was employed to establish a match between the CR and the non-CR group using a caliper width of 0.2 of the standard deviation of the propensity score. Covariate balance was used to determine the quality of PSM according to the standardized difference. All values <0.1 were taken to indicate successful PSM.

A cardiopulmonary exercise test (CPET) was performed within a month of discharge, and an appropriate exercise training program was assigned accordingly.

### Exercise Programs

After the CPET, patients were referred to the outpatient Department of Physical Medicine and Rehabilitation for Phase II CR. The training program for phase II CR followed the recommendations of the American College of Sports Medicine (ACSM) (8). Moderate continuous training with aerobic exercise was prescribed by a board-certified physiatrist who had specialized in CR. The types of exercise were treadmill walking/walking-jogging/jogging, ergometer cycling, stair climbing, or elliptical machine training. The exercise type was adjusted individually for patients with musculoskeletal or neurological disorders. The target intensity of training was 40–60% of peak oxygen consumption (VO_2_/kg), or 10 beats below the heart rate-associated endpoints during the CPET, such as angina, drop in blood pressure, or significant ST segment depression. The training intensity was gradually increased fortnightly to reach the targeted Borg rating of perceived exertion (RPE) of 12–14. The training duration was 40 min, which included 5–10 min of warm-up and cool-down exercises. The training frequency was three sessions per week, with 36 sessions concluding a complete course. Telemetry electrocardiography and oximeter monitors were used for patients with a higher risk, such as LVEF <30%, peak VO_2_/kg <14 ml/kg/min, or significant electrocardiography abnormalities during CPET. We defined high-risk patients for rehabilitation according to the existing literature (9, 10). A total of 10–15 repetitions per set, 1–3 sets per session, and 2–3 non-consecutive days per week of resistance exercise involving both the upper and lower extremities with an RPE intensity of 11–13 was also prescribed after an uneventful moderate continuous training for 4 weeks. The physiatrist also prescribed flexibility exercises for patients as recommended by the ACSM's guideline (8). The ACSM recommends holding each static stretch for 10–30 s. Our patients were advised to maintain hold for at least 15 s with more than four repetitions of each exercise. Exercise compliance after completion of phase II rehabilitation was followed up by the HF nurse. An independent continuation of risk factor modification and exercise was suggested individually.

### Outcomes

The primary outcome was all-cause mortality after discharge, while the secondary outcomes were HF hospitalization after discharge and change in the total 12-item Kansas City Cardiomyopathy Questionnaire short form (KCCQ-12) scores between the 6- and 12-month follow-ups. Life quality assessments were performed using the Chinese version of the KCCQ-12 by the HF nursing specialist at discharge and at the 6- and 12-month follow-ups. Outcome reports and hospitalization data were abstracted from the medical records and phone interviews. The electronic Health Information System was used to confirm the information.

Baseline patient characteristics, clinical HF assessment, risk factors, and GDMT prescriptions, including ACEI or ARB, beta-blockers, MRA, and ARNI, were documented at discharge.

### Statistical Analysis

We used PSM to identify patients who received CR that had similar characteristics to those who did not receive CR. The detailed matching method was described in section Patients.

The chi-square and Mann–Whitney *U*-tests were used to evaluate patient outcomes, including all-cause mortality, HF hospitalization, and total KCCQ-12 score improvement at the 6- and 12-month follow-ups. The CR group was further divided into three groups according to CR frequency (CR ≥ 36 sessions, CR 10–35 sessions, and CR ≤ 9 sessions). We used the chi-square test or Kruskal–Wallis test to compare patients' outcomes between different CR frequency groups. The *post-hoc* test was used for multiple comparisons when the overall difference between groups was significant. Linear-by-linear association and the Jonckheere–Terpstra test were used to determine if there were statistically significant associations between CR frequency and patient outcomes.

Event-free survival relative to all-cause mortality and the first HF rehospitalization before and after PSM in patients in the CR or non-CR groups were calculated using the Kaplan–Meier method and compared using the log-rank test. Cox regression analysis, adjusted for all covariates, was performed to assess the association between CR participation and all-cause mortality, and the association between CR participation and the first HF rehospitalization.

Subgroup analysis was performed to evaluate potential effect modification including age (<75, ≥75 years), sex, HF etiology (ischemic, non-ischemic cardiomyopathy), diabetes mellitus, hypertension, ARNI use, ACEI or ARB use, beta-blocker use, renal function (estimated glomerular filtration rate ≥ 60, <60), ventilatory inefficiency (VE/VCO_2_ slope or VE/VCO_2_ at anaerobic threshold (AT) ≥ 34, < 34), and peak VO_2_/kg level (≥14 ml/kg/min, <14 ml/g/min). CPET parameters were included in the subgroup analysis because of their known associations with HF outcomes (11). The *p-*values for interactions between groups were assessed.

All statistical analyses were performed using SPSS Statistics, Version 22.0 (IBM Corp., Armonk, NY, USA) and R v3.6.1 software. A two-sided *p* < 0.05 was considered statistically significant.

## Results

A total of 792 patients were included in this study and 142 (17.9%) patients attended at least one session of an exercise training program (CR group) within 3 months of discharge. Overall, 571 (72.1%) patients had ischemic cardiomyopathy. There were no differences in patient characteristics, medication, or risk factors after PSM (*n* = 518) between the CR (*n* = 137) and non-CR (*n* = 381) group (Table 1). The baseline peak VO_2_/kg from CPET in the CR and non-CR group was 15.9 ml/kg/min (IQR: 12.85–18.95) and 14.82 (IQR: 11.50–18.57), respectively (*p* = 0.109).

**Table 1:** Patient characteristics before and after matching.

	**Before PSM (*****n*** **= 792)**			**After PSM (*****n*** **= 518)**		
**Variables**	**No cardiac rehabilitation (*n* = 650)**	**Cardiac rehabilitation (*n* = 142)**	**SMD**	**No cardiac rehabilitation (*n* = 381)**	**Cardiac rehabilitation (*n* = 137)**	**SMD**
Age, years; mean (SD)	64.32 (15.03)	57.40 (12.57)	0.500	58.88 (14.43)	57.77 (12.34)	0.083
Sex (male); *n* (%)	463 (71.2)	116 (81.7)	0.248	321 (84.3)	111 (81.0)	0.085
BMI (kg/m^2^); mean (SD)	25.23 (5.05)	26.09 (4.61)	0.178	25.78 (5.02)	26.00 (4.56)	0.046
Systolic BP (mmHg); mean (SD)	124.46 (23.18)	120.34 (20.65)	0.188	120.56 (21.48)	120.37 (20.45)	0.009
Diastolic BP; mean (mmHg) (SD)	72.12 (14.81)	72.18 (14.38)	0.004	71.65 (14.76)	71.78 (13.72)	0.009
HR (beats/min); mean (SD)	81.78 (17.87)	82.68 (15.65)	0.053	81.42 (17.37)	82.37 (15.32)	0.058
Ischemic CM; *n* (%)	466 (71.7)	105 (73.9)	0.051	284 (74.5)	101 (73.7)	0.019
HTN; *n* (%)	425 (65.4)	90 (63.4)	0.042	229 (60.1)	86 (62.8)	0.055
DM; *n* (%)	301 (46.3)	54 (38)	0.168	153 (40.2)	52 (38.0)	0.045
Hyperlipidemia; *n* (%)	260 (40.0)	63 (44.4)	0.088	169 (44.4)	61 (44.5)	0.003
Stroke; *n* (%)	81 (12.5)	19 (13.4)	0.027	48 (12.6)	18 (13.1)	0.016
Hb (gm/dl); mean (SD)	12.73 (2.39	13.34 (2.21)	0.264	13.32 (2.26)	13.34 (2.23)	0.010
eGFR (ml/min/1.73 m^2^); mean (SD)	56.27 (30.78)	65.36 (28.91)	0.305	63.01 (30.12)	65.24 (28.62)	0.076
LVEF; mean (SD)	29.89 (7.10)	29.06 (6.90)	0.119	29.37 (7.27)	29.06 (6.98)	0.042
ACEI or ARB; *n* (%)	535 (82.3)	122 (85.9)	0.099	338 (88.7)	117 (85.4)	0.099
ARNI; *n* (%)	54 (8.3)	13 (9.2)	0.030	27 (7.1)	13 (9.5)	0.087
Beta-blockers; *n* (%)	529 (81.4)	119 (83.8)	0.064	317 (83.2)	115 (83.9)	0.020
MRA; *n* (%)	324 (49.8)	84 (59.2)	0.188	217 (57.0)	80 (58.4)	0.029
Diuretics; *n* (%)	515 (79.2)	109 (76.8)	0.060	297 (78.0)	106 (77.4)	0.014
Atrial fibrillation; *n* (%)	204 (31.4)	84 (59.2)	0.188	109 (28.6)	37 (27.0)	0.036

*PSM, propensity score matching; SMD, standard mean difference; SD, standard deviation; BMI, body mass index; BP, blood pressure; HR, heart rate; CM, cardiomyopathy; HTN, hypertension; DM, diabetes mellitus; Hb, hemoglobin; eGFR, estimated glomerular filtration rate; LVEF, left ventricular ejection fraction; ACEI, angiotensin converted enzyme inhibitor; ARB, angiotensin II receptor blocker; ARNI, angiotensin receptor neprilysin inhibitor; MRA, mineralocorticoid receptor antagonist*.

The median follow-up period was 3.04 (interquartile range [IQR]: 1.72–4.50) years in the PSM cohort (*n* = 518). The all-cause mortality rate was 24.9% and the HF rehospitalization rate was 34.6% during the study period (Table 2A). Patients who underwent the exercise training program at least once (CR group) had a significantly a lower all-cause mortality rate than those who did not undergo the program (non-CR group; 13.9 vs. 28.9%, *p* < 0.001). The HF hospitalization rate was not significantly different between the two groups (35.0 vs. 34.4%, *p* = 0.890; Table 2A). The KCCQ-12 scores revealed significant improvement in quality of life in the CR group compared to the non-CR group at the 6-month follow-up (28.3, IQR: 7.81–45.31 vs. 18.75, IQR: 5.21–36.46; *p* = 0.024). However, it was observed that the quality-of-life improvement was reduced at the 12-month follow-up (28.13, IQR: 7.12–43.40 vs. 20.31, IQR: 4.69–38.19; *p* = 0.084). Table 2B shows the patient outcome comparisons when the CR group was divided into three groups according to CR frequency. The numbers of patients who had CR sessions ≥36, 10–35, and ≤9 were 40 (28.2%), 29 (20.4%), and 73 (51.4%), respectively. The overall difference (all-cause mortality and 1-year improvement in the KCCQ-12 total score) between groups was significant (*p* = 0.002, *p* = 0.025, respectively). In pairwise comparisons with the Bonferroni-adjusted *post-hoc* test, there were significant differences in the all-cause mortality between the CR ≥ 36 and non-CR group (*p* = 0.007). There was a statistically significant increasing trend of higher all-cause mortality with CR frequency fewer groups (*p* < 0.001). There was a statistically significant decreasing trend demonstrating a small improvement in the KCCQ-12 total score in the CR groups with fewer sessions (*p* = 0.018 in 6 months, *p* = 0.029 in 1 year); however, there was no statistically significant difference in the 6-month improvement in the KCCQ-12 total scores between groups (*p* = 0.113).

**Table 2:** Patient outcomes with and without cardiac rehabilitation.

**(A) The cardiac rehabilitation group: patients had at least one session of cardiac rehabilitation**						
**Variables**	**Total (518)**		**Cardiac rehabilitation (137)**	**No cardiac rehabilitation (381)**		***p*** **-value**
All-cause mortality; *n* (%)	129 (24.9%)		19 (13.9%)	110 (28.9%)		<0.001
HF rehospitalization; *n* (%)	179 (34.6%)		48 (35.0%)	131 (34.4%)		0.890
KCCQ-12 total scores, 6-month improvement; median (IQR)	20.83 (5.69–39.84) (*n* = 362)		28.13 (7.81–45.31) (*n* = 115)	18.75 (5.21–36.46) (*n* = 247)		0.024
KCCQ-12 total scores, 1-year improvement; median (IQR)	23.96 (5.56–40.63) (*n* = 363)		28.13 (7.12–43.40) (*n* = 120)	20.31 (4.69–38.19) (*n* = 243)		0.084
**(B) The cardiac rehabilitation group was divided into three groups according to CR frequency**						
**Variables**	**Cardiac rehabilitation (137)**			**No cardiac rehabilitation (381)**	***p*** -value^*^	***p*** **for trend**^†^
	**CR ≥36 (*****n*** **= 40)**	**CR = 10–35 (*****n*** **= 27)**	**CR ≤9 (*****n*** **= 70)**			
All-cause mortality; *n* (%)	2 (5%)^a^	6 (22.2%)	11 (15.7%)	110 (28.9%)^a^	0.002	<0.001
HF rehospitalization; *n* (%)	12 (30.0%)	8 (29.6%)	28 (40.0%)	131 (34.4%)	0.664	0.684
KCCQ-12 total scores, 6-months improvement; median (IQR)	31.51 (11.98–45.70) (*n* = 36)	30.73 (6.64–46.35) (*n* = 24)	22.92 (7.29–44.79) (*n* = 55)	18.75 (5.21–36.46) (*n* = 247)	0.113	0.018
KCCQ-12 total scores, 1-year improvement; median (IQR)	32.99 (16.79–47.13) (*n* = 38)	32.64 (20.40–45.92) (*n* = 21)	18.75 (4.69–38.02) (*n* = 61)	20.31 (4.69–38.19) (*n* = 243)	0.025	0.029

*CR, cardiac rehabilitation; HF, heart failure; KCCQ-12, Kansas City Cardiomyopathy Questionnaire short form 12; IQR, interquartile range*.

* *To use chi-square test or Kruskal–Wallis test to compare patients' outcome (all-cause mortality, HF hospitalization, and KCCQ-12 score improvement) between different CR frequency groups*.

a *The post-hoc test was used for multiple comparisons when overall difference between groups was significant*.

† *Linear-by-linear association and the Jonckheere–Terpstra test were used to determine if there were statistically significant linear trend between CR frequency on patients' outcome*.

**Figure 1:**
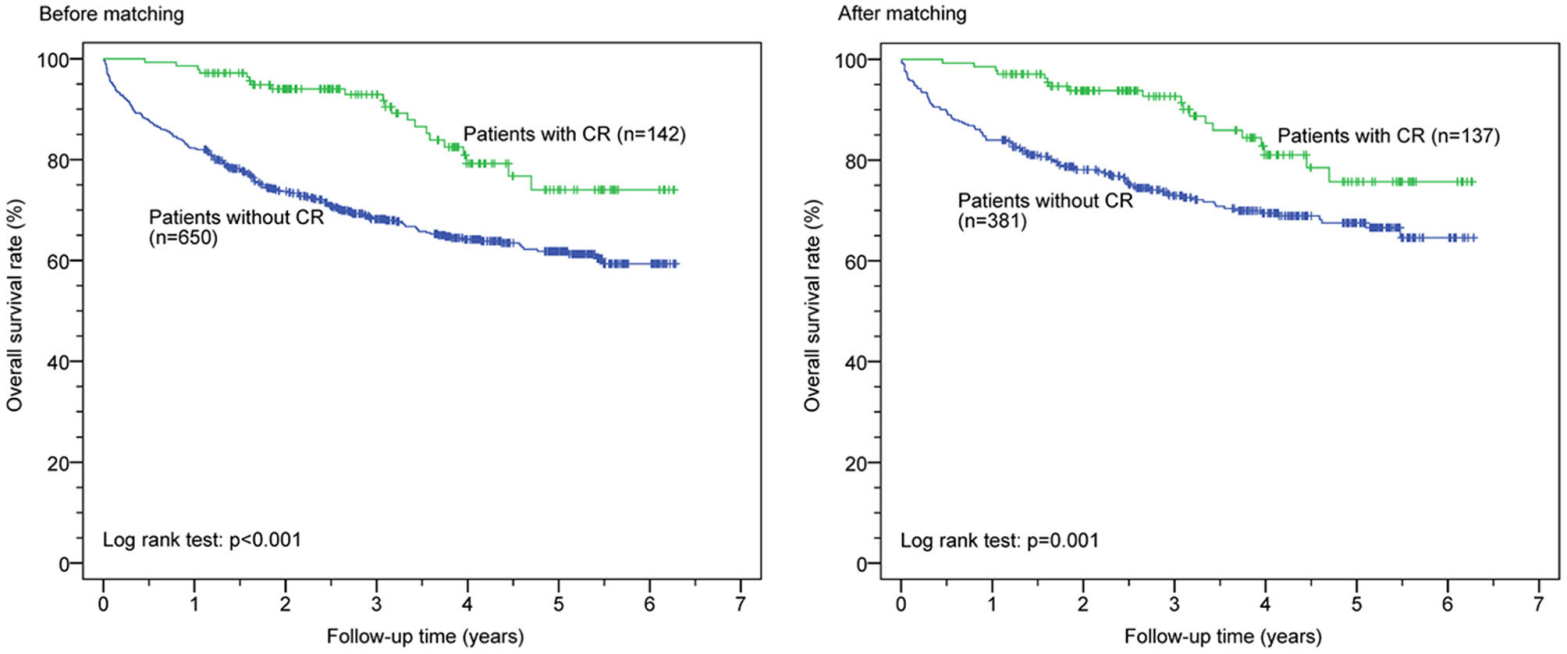
Cardiac rehabilitation (CR) and all-cause mortality before and after propensity score matching.

**Figure 2:**
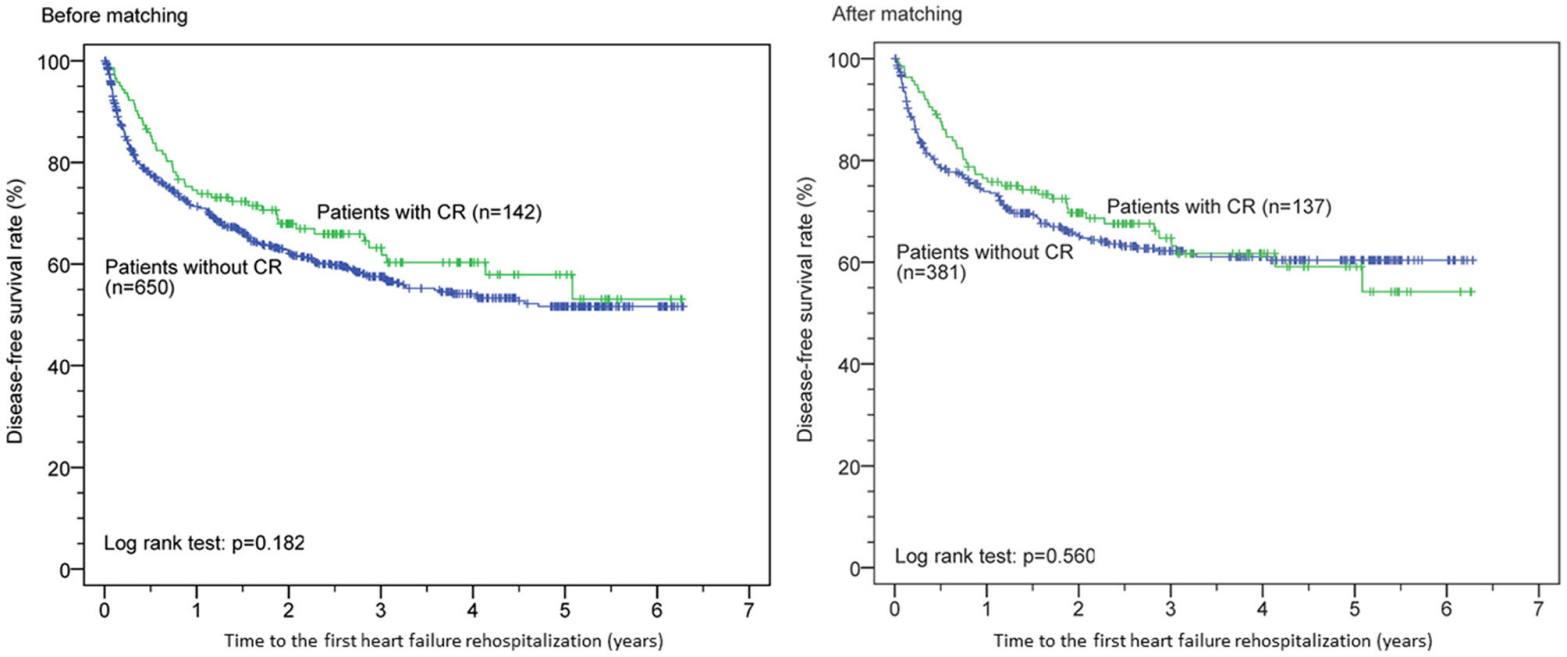
Cardiac rehabilitation (CR) and time to the first heart failure rehospitalization before and after propensity score matching.

Kaplan–Meier survival curves before and after PSM for both groups are shown in Figure 1. CR was associated with significantly reduced all-cause mortality before (*p* < 0.0001) and after (*p* < 0.001) PSM but was not significantly associated with reduced first HF rehospitalization before (*p* = 0.182) and after (*p* = 0.560) PSM (Figure 2). Additionally, the Cox proportional hazard analysis revealed that the CR group had a significantly lower all-cause mortality than the non-CR group (hazard ratio [HR]: 0.490 [95% confidence interval {CI}: 0.308–0.778], *p* = 0.003) after adjusting all covariates used to generate the propensity score. Likewise, the association between the rate of HF rehospitalization and CR was not significant (HR: 0.911 [95% CI: 0.670–1.239], *p* = 0.553) in this study.

**Figure 3:**
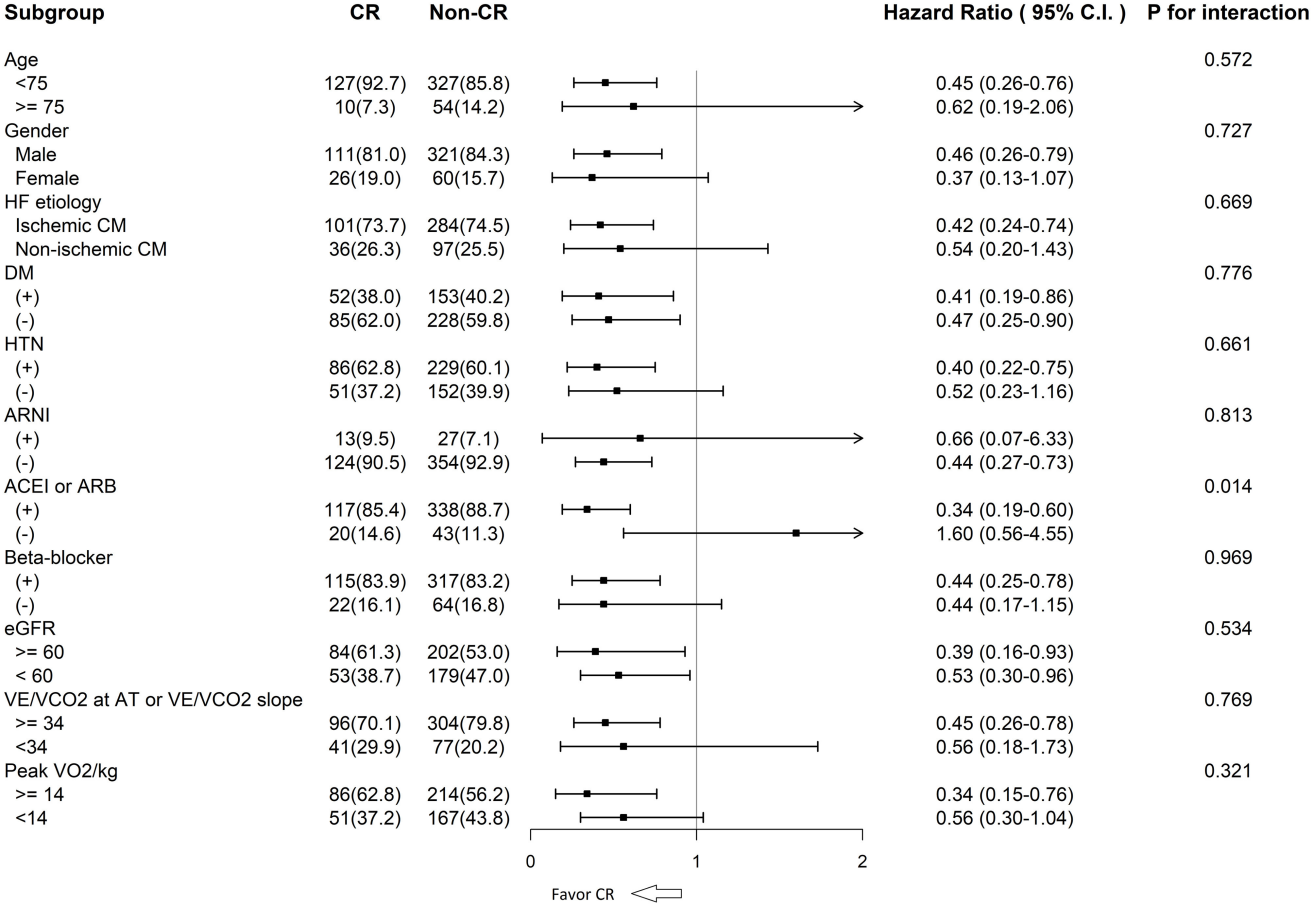
CR and all-cause mortality among the subgroups. CR, cardiac rehabilitation; HF, heart failure; CM, cardiomyopathy; DM, diabetes mellitus; HTN, hypertension; ARNI, angiotensin receptor neprilysin inhibitor; ACEI, angiotensin converted enzyme inhibitor; ARB, angiotensin II receptor blocker; eGFR, estimated glomerular filtration rate; VE/VCO_2_, ventilatory equivalent for carbon dioxide; AT, anaerobic threshold; VO_2_/kg, oxygen consumption per kilogram.

In the subgroup analysis, the CR group was found to have better clinical outcomes (all-cause mortality) regardless of age, sex, HF etiology, diabetes mellitus, hypertension, estimated glomerular filtration rate estimated glomerular filtration rate, ventilation inefficiency, peak VO_2_/kg, and prescribed medications (Figure 3). However, the CR groups had a lower risk of total mortality compared with the non-CR groups in patients who had ACEI or ARB prescriptions (HR: 0.34 [95% CI: 0.19–0.60]), but not in those without the prescriptions (HR: 1.60 [95% CI: 0.56–4.55]; interaction ACEI/ARB prescriptions^*^CR *p* = 0.014). In the non-CR groups, ACEI or ARB treatment did not have an impact on patient outcomes (*p* = 0.258, 95% CI: 0.749–2.931).

## Discussion

This study revealed that multidisciplinary CR was associated with a better prognosis in patients who were just discharged from hospital for acute HF. The significant effect in decreased all-cause mortality was evident even after PSM for covariates related to HF prognosis. Compared with that of the non-CR group, the quality-of-life improvement of the CR group was significant at 6 months but not in 1 year. The placebo effect should be considered (12). The dose-response association was noted in the survival rate. The higher adherence to CR implied better outcomes. Furthermore, the subgroup analysis demonstrated an obvious association between exercise training and clinical outcome. However, in contrast to patients who had ACEI/ARB prescriptions, the primary outcomes for the CR and non-CR group were similar in patients without ACEI/ARB prescriptions.

Recently, a Cochrane systematic review reported that CR may make little or no difference in short-term (<12 months follow-up) all-cause mortality (27 trials, risk reduction [RR]: 0.89 [95% CI: 0.66–1.21]) (13). CR showed a slight reduction trend in all-cause mortality over a follow-up period of 12 months, mostly based on the large HF-ACTION study (RR: 0.88 [95% CI: 0.75–1.02]). In the HF-ACTION study, patients could not be enrolled until 6 weeks after HF hospitalization (1). However, the recently published REHAB-HF trial (Rehabilitation Therapy in Older Acute Heart Failure Patients) showed that an early, transitional, tailored, progressive rehabilitation intervention resulted in greater improvement in physical-function domains than usual care (14). As such, the medium- to long-term survival benefits of an earlier enrollment of patients (i.e., just discharged from the hospital) for CR are still unknown. As demonstrated in our study, patients who were discharged from hospital after HF had significant survival benefits when they received CR within 3 months.

The Cardiac Rehabilitation Outcome Study in Heart Failure, a systematic review and meta-analysis, including patients with HF and reduced EF (HFrEF; 25 randomized control trials, 4,481 individuals), showed that exercise-based CR was not associated with lower mortality and hospitalization (15); instead, it was only associated with a better quality of life and exercise capacity (15). Multidisciplinary CR seems to be better than exercise-based CR. A Japanese multidisciplinary CR study that enrolled patients hospitalized for acute HF revealed that comprehensive interventions, including exercise and patient education, were associated with a lower risk of all-cause mortality and HF rehospitalization (16).

The modern CR program should be multidisciplinary and comprise three distinct phases: inpatient, outpatient, and in the community/home (17). Multidisciplinary CR that includes dietary counseling, optimization of medications, psychosocial support, smoking cessation, and exercise training is possibly the key to improving clinical outcomes. Education provided by HF nurses was discovered to affect CR enrollment, adherence, and completion (18–20). CR with the core components of a disease management program could boost the effect of exercise training (21, 22).

To the best of our knowledge, this is the first study to show an interaction effect of renin-angiotensin-aldosterone system (RAAS) inhibitors on the CR program. While exercise training may result in post-myocardial infarction scar thinning, no additional scar thinning was observed in the post-infarct myocardium of patients receiving both exercise training and RAAS inhibitors (23). While exercise training may also cause plaque erosion and trigger acute coronary syndrome (24), RAAS inhibitors may have a protective effect against plaque rupture leading to myocardial infarction (25). Hence, the benefit provided by exercise could be diminished in those without RAAS inhibitor prescriptions resulting in similar outcomes between CR and non-CR group. The contributing predictors for a beneficial CR effect may include concomitant prescription of RAAS inhibitors and a multidisciplinary rehabilitation program.

Physical exercise has two opposite pathway responses in the RAAS. One is the classical angiotensin-converting enzyme (ACE), angiotensin II, and angiotensin type I receptor pathway, which is associated with vasoconstriction, myocardial hypertrophy, and sodium/water retention. The other one is the counter-regulatory or vasodilator pathway involving ACE2, angiotensin-(1-7), and the Mas receptor, which is associated with anti-hypertrophy, cardioprotective, and reno-protective effects (26, 27).

Acute exercise may induce elevations in plasma renin activity, aldosterone levels, and vasopressin levels (28). In response to acute exercise, ACE levels increase independent of the ACE genotype (29), and its serum (ACE and ACE2) activities may be involved in the pathomechanism of HF (30, 31). RAAS inhibitors block the first pathway, which is induced by acute exercise (32), while physical training activates the second pathway (30, 33); collectively, they translate to a better clinical outcome in the CR program. In contrast, the clinical outcomes of the CR and non-CR groups were similar for patients without RAAS inhibitor prescriptions.

For decades, the implementation of the CR program in patients with HFrEF has been challenging (34). Patients with HFrEF, especially those discharged from the hospital, are prone to arrhythmias, hemodynamic instability, and fluid overload, and are often older and frailer. As such, the skepticism about CR is magnified by safety concerns. Additionally, the CR program is not lucrative for hospitals or cardiologists compared to other intervention procedures. Finally, a CR program is a slow intervention where the endpoint benefits usually take longer to be realized (35). The benefits from coronary interventions or cardiovascular surgeries are usually immediate and obvious. As this study highlighted a significant reduction in all-cause mortality in the CR group, efforts should be made to facilitate a CR program so that more patients can participate and benefit from it.

Another finding of our study indicated that CR had no significant effect on rehospitalization. A potential explanation for this finding might be that the discharged HF population belonged to a relatively high-risk group which tended to be readmitted. More than one-third of patients experienced HF rehospitalizations (CR: 35.0%, vs. non-CR: 34.4%). In contrast, the Empagliflozin Outcome Trial in Patients with Chronic Heart Failure with Preserved Ejection Fraction (EMPEROR-Preserved) trial showed that HF rehospitalization occurred in 8.6% in the empagliflozin group and in 11.8% in the placebo group (HR: 0.71; 95% CI, 0.60–0.83) (36). Their readmission rate was much lower than that in our study population.

This study had a few limitations. First, it was a retrospective observational study. Nevertheless, real-world data instead of data from well-designed randomized control trials were reviewed. Second, only inpatients with HFrEF were enrolled, and thus, there may be selection bias due to the exclusion of outpatients with HFrEF and those with HF and preserved EF. Third, the frequency of the CR sessions varied from one time to more than 36 times. However, it should be emphasized that the benefits of the CR program could still be seen despite such heterogeneity. Fourth, we did not use cardiovascular mortality as one of our study endpoints because attempting to classify the cause of death is problematic. Instead, all-cause mortality is a clinically relevant, objective, and unbiased endpoint. Fifth, being a retrospective study and not designed to evaluate the interactions of prescribed drugs with CR, the fact that the benefit of CR was decreased in patients who were not on RAAS inhibitors warrants evaluation. Finally, the study population was small compared to large multi-center or international registries. Nonetheless, an interaction effect between RAAS inhibitors and the CR program was discovered for the first time. Moreover, baseline CPET parameters were also used for subgroup analysis; these have not been used in other studies.

Further, neurohormonal assays and protein expression analyses, with or without RAAS inhibitors, in patients with HF in the CR program are needed to further validate the findings. The sodium-glucose co-transporter 2 was added to the ESC Guidelines for the treatment of chronic HFrEF in 2021. The potential interaction between sodium-glucose co-transporter 2 and the benefit of CR should be evaluated in the future. Moreover, larger, prospective evaluation is necessary.

In summary, post-discharge CR participation was associated with reduced medium- to long-term all-cause mortality and improved the quality of life in the first 6 months in patients with HFrEF. The association between CR participation and HF readmission, however, was not significant. In contrast to patients who received RAAS inhibitors, the primary outcomes in the CR and non-CR group were similar for patients who did not receive RAAS inhibitors. Predictors for successful CR may include multidisciplinary rehabilitation and prescription of RAAS inhibitors.

Overall, this study provides evidence of the benefits of CR as a standard treatment in patients with post-acute HF. Hence, efforts should be made to implement CR with eligible patients.

## Data Availability Statement

The original contributions presented in the study are included in the article/supplementary material, further inquiries can be directed to the corresponding author/s.

## Ethics Statement

The studies involving human participants were reviewed and approved by Chang Gung Medical Foundation Institutional Review Board. Written informed consent for participation was not required for this study in accordance with the national legislation and the institutional requirements.

## Author Contributions

S-MC: conceptualization, writing—original draft, review and editing, and supervision. L-YW and Y-LC writing—original draft and data curation. M-YL, C-LW, A-NC, and T-LS: data curation and investigation. P-JW, CC, T-HY, and J-KC: data curation. P-CC and M-KW: investigation. C-IC: visualization and validation. All authors contributed to the article and approved the submitted version.

## Funding

This study was supported by a program grant from the Chang Gung Medical Foundation (Grant Numbers: CMRPG8J1441 and CMRPG8L0531).

## Conflict of Interest

The authors declare that the research was conducted in the absence of any commercial or financial relationships that could be construed as a potential conflict of interest.

## Publisher's Note

All claims expressed in this article are solely those of the authors and do not necessarily represent those of their affiliated organizations, or those of the publisher, the editors and the reviewers. Any product that may be evaluated in this article, or claim that may be made by its manufacturer, is not guaranteed or endorsed by the publisher.
